# Effects of Solvent Vapor Annealing on Morphology and Charge Transport of Poly(3-hexylthiophene) (P3HT) Films Incorporated with Preformed P3HT Nanowires

**DOI:** 10.3390/polym12051188

**Published:** 2020-05-22

**Authors:** Mingu Jang, Yang-Il Huh, Mincheol Chang

**Affiliations:** 1 Department of Polymer Engineering, Graduate School, Chonnam National University, Gwangju 61186, Korea; god9686@naver.com; 2 School of Polymer Science and Engineering, Chonnam National University, Gwangju 61186, Korea; 3 Alan G. MacDiarmid Energy Research Institute, Chonnam National University, Gwangju 61186, Korea

**Keywords:** solvent vapor annealing, poly(3-hexylthiophene), molecular ordering, morphology, organic field-effect transistors

## Abstract

We systematically studied the influence of solvent vapor annealing on the molecular ordering, morphologies, and charge transport properties of poly(3-hexylthiophene) (P3HT) thin films embedded with preformed crystalline P3HT nanowires (NWs). Solvent vapor annealing (SVA) with chloroform (CF) was found to profoundly impact on the structural and morphological changes, and thus on the charge transport characteristics, of the P3HT-NW-embedded P3HT films. With increased annealing time, the density of crystalline P3HT NWs was increased within the resultant films, and also intra- and intermolecular interactions of the corresponding films were significantly improved. As a result, the P3HT-NW-embedded P3HT films annealed with CF vapor for 20 min resulted in a maximized charge carrier mobility of ~0.102 cm^2^ V^−1^ s^−1^, which is higher than that of pristine P3HT films by 4.4-fold (μ = ~0.023 cm^2^ V^−1^ s^−1^).

## 1. Introduction

In recent years, solution-processible conjugated polymers have garnered a great deal of attention for the practical printing of organic field-effect transistors (OFETs), organic photovoltaics, and interface materials due to their low cost and large-area processability [1,2,3,4,5,6]. Typically, they have been utilized and studied as active materials for OFETs because of their semiconducting properties, arising from the extended π-conjugation of unhybridized P_z_ carbon orbitals along the backbone [7]. However, OFETs based on conjugated polymers commonly exhibit a low charge transport due to the low degree of intra- and intermolecular ordering (i.e., low crystallinity) in semiconductor films, resulting in significant limitations on their practical commercialization [8,9,10,11]. In general, semicrystalline polymer films are generated via solution processing of conjugated polymers; small crystallites are randomly distributed within a largely amorphous matrix [12,13]. This morphological feature is apt to involve grain boundaries and trap sites that prevent efficient charge hopping between crystalline domains [14,15].

To date, thermal annealing has been commonly used as a representative approach to increase the degree of crystallinity, and thus the charge carrier mobility, of conjugated polymer thin films for high performance OFETs. While this approach is quite effective, it leads to degradation of the favorable properties of the conjugated polymer itself and is somewhat limited in achieving a high degree of crystallinity [16,17]. Recently, an alternative approach, solvent vapor annealing (SVA), has been preferably used to improve the crystallinity and concomitant charge transport of conjugated polymer thin films because of its ease of use and outstanding effectiveness at room temperature [18,19]. In a typical process, solvent molecules penetrate into a conjugated polymer thin film at room temperature and increase the movement of polymer chains by their ability to serve as a plasticizer, resulting in increased crystallinity of resultant films [20,21,22].

More recently, conjugated polymer nanowires (NWs) that are preaggregated in solutions via a solution treatment, such as sonication or ultraviolet (UV) irradiation, have been incorporated into conjugated polymer films to enhance intra- and intermolecular interactions (i.e., crystallinity) of polymer chains [23,24]. In general, strong noncovalent π–π interactions between polymer chains lead to the nucleation and growth of one-dimensional (1-D) crystalline NWs. Accordingly, charge transport favorably occurs along the direction of the long axis of the NWs [25]. So far, several reports have demonstrated successful enhancement in molecular ordering and charge transport of a conjugated polymer thin film by the incorporation of the 1-D NWs of the corresponding polymer [9,26,27,28,29,30]. Post-solvent vapor annealing is envisioned to further enhance molecular ordering of NW-embedded conjugated polymer thin films commensurate with efficient charge transport. However, there are few reports that study the solvent vapor annealing of conjugated polymer films with the incorporation of preformed crystalline NWs [31,32,33,34].

Herein, we systematically study SVA of poly(3-hexylthiophene) (P3HT) thin films embedded with preformed crystalline P3HT NWs and demonstrate that SVA can improve the molecular ordering and concomitant charge transport properties of the resultant P3HT films, resulting in the additional formation of crystalline P3HT NWs within the corresponding films. It was discovered that 20 min of chloroform (CF) vapor annealing can result in a maximized charge carrier mobility of the P3HT-NW-embedded P3HT films, which is highly superior compared to those of pristine P3HT films with or without P3HT NWs. The influence of SVA on the molecular ordering, morphologies, and charge transport characteristics of the P3HT-NW-embedded P3HT films was investigated using UV–VIS absorption spectroscopy, atomic force microscopy (AFM), polarized optical microscopy (POM), and grazing incident X-ray diffraction (GIXRD), and charge carrier mobility measurements. Further, the correlation between the solvent-annealing parameters, molecular ordering, morphologies, and charge transport characteristics of the resultant films is elucidated.

## 2. Materials and Methods

Materials: Poly(3-hexylthiophene) with a regioregularity of 96% and a molecular weight of 51 kDa was purchased from Rieke Metals Inc. (Lincoln, NE, USA). Chloroform (anhydrous grade) was purchased from Sigma-Aldrich (Milwaukee, WI, USA). All chemicals were used without further purification.

Preparation of P3HT NWs in Solution: The P3HT solutions were prepared by mixing ten milligrams (10 mg) of P3HT into 2 mL of chloroform, followed by heating at 55 °C for 60 min for complete dissolution. To generate P3HT NWs in solution, the as-prepared P3HT solutions were treated by UV irradiation, following the procedure reported in previous literature [24].

OFET Fabrication and Solvent Vapor Annealing: The FET devices with bottom-gate bottom-contact geometries were manufactured by following the reported procedure [35]. All P3HT films were coated onto precleaned devices via spin-coating at a rate of 2000 rpm for 60 s in ambient condition. Subsequently, the as-prepared OFET devices were placed on a polytetrafluoroethylene (PTFE) platform (3 cm × 3 cm × 1 cm) in a sealed glass container (7 cm in diameter and 2 cm in height) filled with 12 mL CF, and then annealed with CF vapor for a specified time at room temperature. Finally, the as-annealed devices were placed in a vacuum chamber (1 Torr) overnight at 55 °C for the removal of the residual solvent.

Characterizations: A Keithly 4200 semiconductor analyzer (Keithley, Cleveland, OH, USA) was utilized to test the OFET devices in a N_2_ filled glovebox. The charge mobility was calculated in the saturation regime at a drain-source voltage (V_DS_) of −80 V and a gate-source voltage (V_GS_) of −80 V [21,22]. UV–VIS spectra of P3HT films were recorded by using a UV–VIS spectrometer (Optizen 2120, Mecasys, Daejeon, Korea). Polarized optical microscopy (POM) images were obtained using a LEICA DM 7500PT with an iCM 3.0 IMT i-Solution Inc. digital camera (i-Solution Inc., Seongnam, Korea). For UV–VIS and POM characterization, P3HT thin films were spin-coated on a precleaned glass substrate. Grazing incidence X-ray diffraction (GIXRD) data were collected using a PANalytical X’pert Pro system (Cu X-ray source operating at 45 kV and 40 mA, Malvern Panalytical, Malvern, United Kingdom). The grazing-incidence angle was fixed at 0.2° and the detector was scanned from 3 to 20°. The surface morphologies of P3HT films were measured by using an atomic force microscopy (XE-100, park systems, Suwon, Korea) operated in tapping mode with a silicone tip (OMCL-AC160TS, park systems, Suwon, Korea).

## 3. Results and Discussion

**Figure 1:**
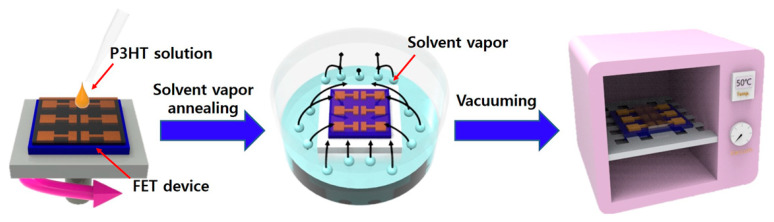
Schematic illustration of the procedure for solvent vapor annealing of poly(3-hexylthiophene)-nanowire (P3HT-NW)-embedded P3HT thin films spin-coated on a field-effect transistor (FET) device substrate.

To study the influence of SVA on the molecular ordering, morphologies, and charge transport properties of conjugated polymer films embedded with corresponding polymer NWs, P3HT was used as a model polymer because of its good solution processability, its self-assembly into crystalline structures, and its good charge transport characteristics [36,37,38]. The P3HT solutions containing 1-D crystalline P3HT NWs were prepared by UV irradiation, following the procedures reported in previous literature [24]. The SVA of the P3HT-NW-embedded P3HT films was performed as illustrated in Figure 1. Specifically, a FET device substrate deposited with a P3HT-NW-embedded P3HT film via spin-coating was placed into a sealed glass bottle which contained CF and was then annealed for various times (1, 3, 5, 10, and 20 min) at ambient atmosphere. SVA using CF was performed at room temperature, rather than at higher temperatures, because of its ease of control and cost-effectiveness. SVA at room temperature is efficient enough to promote the movement of polymer chains and, thus, crystallization when a volatile solvent is used [18,19]. After SVA, the residual solvent molecules were moved to a vacuum oven at 55 °C overnight. Due to its good solubility to P3HT and high volatility at room temperature, CF was used as the atmosphere vapor for SVA of the P3HT films.

**Figure 2:**
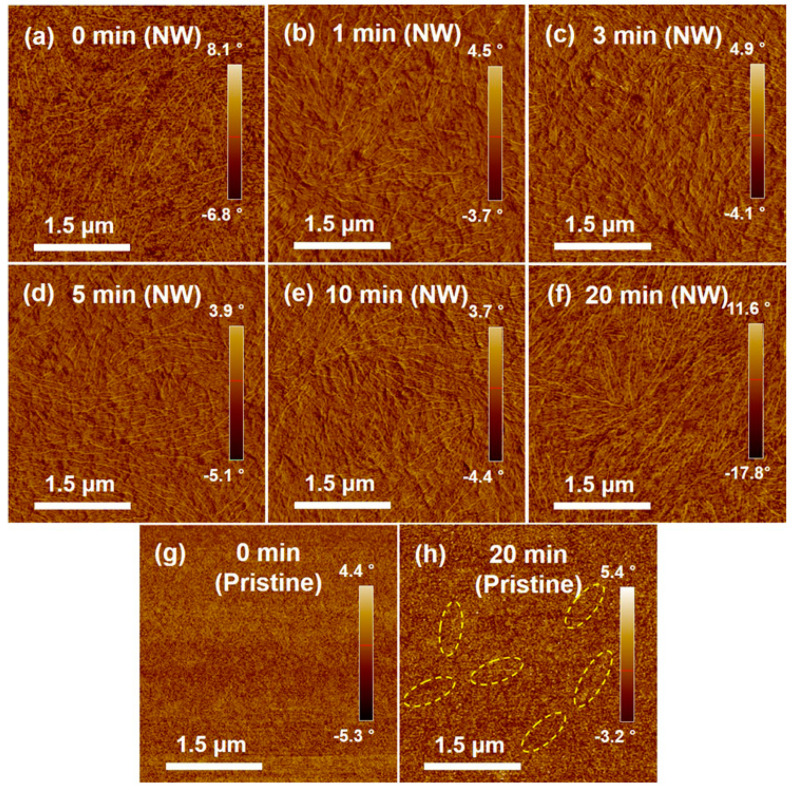
Tapping-mode atomic force microscopy (AFM) images of spin-coated P3HT-NW-embedded P3HT thin films annealed by chloroform (CF) vapor for (**a**) 0, (**b**) 1, (**c**) 3, (**d**) 5, (**e**) 10, and (**f**) 20 min, and spin-coated pristine P3HT films annealed by CF vapor for (**g**) 0 and (**h**) 20 min. The dotted yellow circle surrounds one of the NW-like P3HT structures.

**Figure 3:**
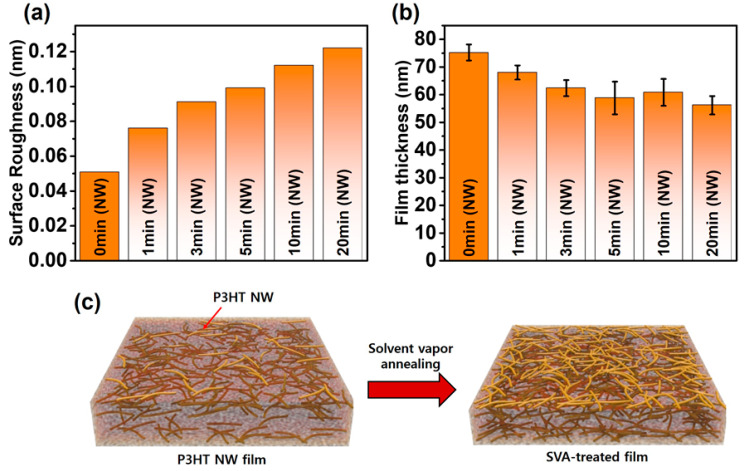
(**a**) Surface roughness and (**b**) film thickness of P3HT-NW films annealed by CF vapor for different times (0, 1, 3, 5, 10, and 20 min). (**c**) Schematic illustration of the influence of solvent vapor annealing (SVA) on the morphology of P3HT films. The colored line depicts the P3HT NW.

Atomic force microscopy (AFM, Park Systems, Suwon, Korea) was employed to investigate the impact of SVA on the morphology of P3HT films embedded with crystalline P3HT NWs preformed in solution (Figure 2). The pristine P3HT films spin-coated from a solution containing P3HT NWs clearly exhibited randomly distributed P3HT NWs that are preformed aggregates in solution via UV irradiation, as shown in Figure 2a. As the annealing time increased up to 20 min, the density of NW structures apparently increased, as shown in Figure 2b–f. This result demonstrates that SVA facilitates the formation of P3HT-NW structures. Note that further increasing the time up to 50 min did not lead to discernable changes in the morphology of the annealed films (Figure S1 from the Supplementary Materials). It is believed that the formation of P3HT NWs resulted from the rearrangement of P3HT chains via favorable π–π interactions; CF molecules that penetrate into the P3HT films act as a plasticizer and thus increase the mobility of the P3HT single chains within the films. Similarly, formation of NW aggregates was observed in the pristine P3HT films that were spin-coated from a solution containing no P3HT NWs upon SVA (Figure 2g,h); the morphology of the pristine P3HT films was seen to be amorphous and featureless. However, the formation of P3HT NWs was less prominent in the pristine P3HT films than in the P3HT films embedded with preformed P3HT NWs. The nucleation sites and small crystallites would be more abundant in the latter films because UV irradiation treatment of P3HT solutions facilitates the nucleation and growth of P3HT NWs [8,24]. Accordingly, the formation of P3HT NWs would be far more promoted by nucleation sites and small crystallites upon SVA in the P3HT films spin-coated from UV-treated solutions than in the P3HT films spin-coated from pristine solutions. Conceivably, the surface roughness of the P3HT-NW-embedded P3HT films was changed after SVA. The root-mean square (RMS) gradually increased up to ~0.12 nm with increasing SVA time, while the RMS of the pristine P3HT films with P3HT NWs was recorded to be ~0.05 nm (Figure 3a and Figure S2). Interestingly, the film thickness was markedly decreased from 75 to 56 nm by SVA as the annealing time increased (Figure 3b). As illustrated in Figure 3c, the increased roughness is attributed to the increased quantity of NW structured aggregates, while the decreased volume is led by the increased crystallinity, as the film structure changes from an amorphous to a crystalline state. Generally, the crystallinity of a polymer film increases as the packing distance between polymer chains decreases, indicative of a decreased volume of the film [8,39,40].

**Figure 4:**
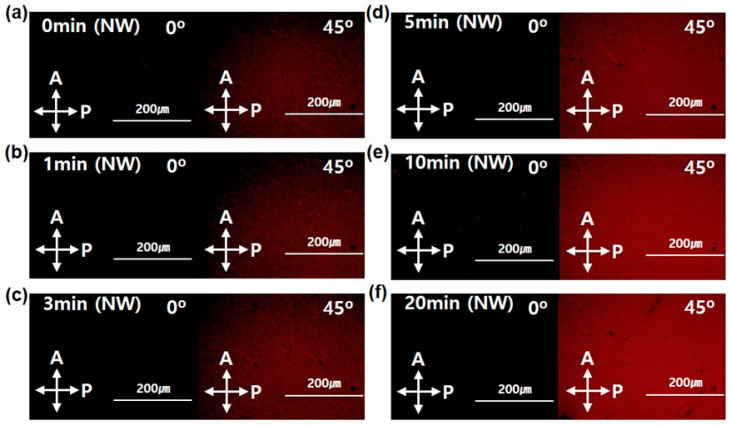
Polarized optical microscopy (POM) images of P3HT-NW films annealed by CF vapor for different times: (**a**) 0, (**b**) 1, (**c**) 3, (**d**) 5, (**e**) 10, and (**f**) 20 min.

The observation of morphological change in the P3HT-NW-embedded P3HT films was also performed using polarized optical microscopy (POM), as shown in Figure 4. With an increased SVA time, a significant change in the birefringent texture of the films was observed through crossed polarizers. While the pristine P3HT-NW-embedded films showed slightly bright features arising from the ordered NWs preformed by UV irradiation of the solution, the films that were CF vapor annealed for longer exhibited increasingly bright birefringent textures. This observation of improved birefringence is another indicator for enhanced molecular ordering, namely, crystallinity of the resultant films [8,24].

**Figure 5:**
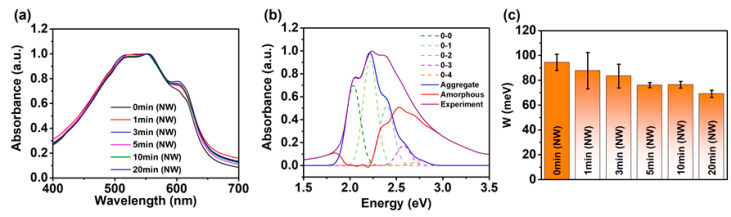
(**a**) Normalized UV–VIS absorption spectra of P3HT-NW films annealed with CF vapor for 1, 3, 5, 10, and 20 min. (**b**) Absorption spectrum of P3HT-NW films annealed with CF vapor for 20 min deconvoluted by Spano analysis using Equation (1). The red line indicates the spectrum of amorphous P3HT chains, and the blue line depicts the spectrum of P3HT aggregates in the film. (**c**) Calculated exciton bandwidth (*W*) of P3HT-NW-film CF vapor annealed for various times.

Figure 5a displays the UV–VIS absorption spectra of the P3HT-NW-embedded P3HT films annealed by CF vapors for various times ranging from 0 to 20 min, which can elucidate the effect of SVA on the molecular-scale structure (i.e., intra- and intermolecular interactions) of the polymer thin films [24,41,42,43]. The higher energy band (π–π* intraband transition) observed at ~529 nm is related to the intrachain states in single P3HT chains with a random-coil conformation, while the lower energy features (vibronic bands) that appear at ~553 and 602 nm are associated with interchain coupling in ordered P3HT aggregates. As the annealing time increased up to 20 min, the lower energy features were increasingly developed compared to the higher energy features, indicative of enhanced intermolecular interactions between P3HT chains of the corresponding films. The same trend was observed in the pristine P3HT films upon CF vapor annealing, as shown in Figure S3 (from the Supplementary Materials). This result implies that annealing with CF vapors promotes the formation of ordered structures via favorable π–π interactions, relative to amorphous P3HT structures, which is consistent with the AFM data shown in Figure 2. It should be noted that an increased annealing time of up to 50 min did not result in further enhancement in the lower energy features relative to the higher energy features (Figure S4 from the Supplementary Materials).

The intramolecular ordering of P3HT chains was further analyzed by the combination of quantitative modeling and UV–VIS absorption spectroscopy of the P3HT films (Figure 5b, Figures S5 and S6) [24,44]. The theoretical vibronic absorption of the ordered P3HT structures is defined by Equation (1) and is fitted to the experimental spectra of P3HT films to obtain the free exciton bandwidth (*W*). The *W* value is inversely proportional to the intramolecular ordering, which is calculated using the intensities of the (0–0) and (0–1) transitions that correspond to the peaks at ~602 and 553 nm, respectively.

(1) $$A\propto \sum_{m=0}\left(\frac{e^{-S}S^{m}}{m!}\right)\times {\left(1-\frac{We^{-S}}{2E_{P}}G_{m}\right)}^{2}\times \exp\left(-\frac{{\left(E-E_{0–0}-mE_{P}-1/2WS^{m}e^{-S}\right)}^{2}}{2\sigma^{2}}\right)$$

where *A* is the absorbance, *S* is the Huang–Rhys factor (~1.0), *E_p_* is the intermolecular vibrational energy of the symmetric vinyl stretching mode (~0.18 eV), *σ* is the Gaussian linewidth, *G_m_* is a constant that depends on the vibrational level *m* (e.g., *m* = 0 for the (0–0) transition) as defined by *G_m_* = ∑*n*(≠*m*)*S^n^*/*n*!(*n* − *m*), where *n* is the vibrational quantum number, S is the Huang-Rhys factor [35,44,45]. Solvent vapor annealed P3HT films and P3HT-NW-embedded P3HT films showed relatively lower *W* values compared to their pristine films (Figure 5c, Figures S3 and S4). For instance, the *W* values of pristine P3HT films and pristine P3HT-NW-embedded P3HT films are ~107.8 and 94.5 meV, respectively. However, a significant decrease in *W* was led by CF vapor annealing for 20 min for both films (~94.5 for the former film and 69.1 meV for the latter film, respectively). These results indicate that CF vapor annealing would effectively improve the intramolecular ordering of the resultant P3HT films.

**Figure 6:**
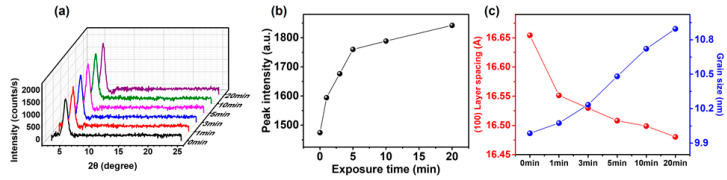
(**a**) Grazing incidence X-ray diffraction (GIXRD) profiles of P3HT-NW films annealed with CF vapor for various times (i.e., 0, 1, 3, 5, 10, and 20 min). (**b**) (100) peak intensity of P3HT-NW films as a function of annealing time. (**c**) Plots of the corresponding (100) layer spacing (left axis) and crystal grain size (right axis) along the (100) direction.

The changes in intra- and intermolecular interactions of P3HT chains can be mirrored by the changes in film crystallinity [8,24]. Figure 6a shows X-ray diffractorgrams acquired from GIXRD measurements of the P3HT films. The intensity of the characteristic (100) peak at 2θ = 5.23–5.43°, which is associated with the lamellar packing of P3HT, gradually increased with increasing annealing time (Figure 6b) [46,47]. This increase is attributed to an increased number of crystalline P3HT NWs formed by CF vapor annealing. Figure 6c shows the evolution of lamellar spacing and grain size as a function of the annealing time. For example, the 2θ value of the P3HT-NW-embedded P3HT films was changed from 5.30° to 5.36° after 20 min CF vapor annealing, indicative of a decrease in d-spacing of the (100) plane from 16.65 to 16.48 Å and suggestive of enhanced interdigitation between the alkyl side chains or increased side chain tilt. In contrast to the lamellar spacing, the average coherence length (i.e., grain size) corresponding to P3HT lamellar packing was increased upon the CF vapor annealing; the P3HT-NW-embedded film grain size changed at a value of 10.0 to 10.9 nm after 20 min CF vapor annealing (Figure 6c). The grain size was calculated by the Scherrer formula, in which the average grain size correlates with the full width at half-maximum (fwhm) of the (100) peak.

**Figure 7:**
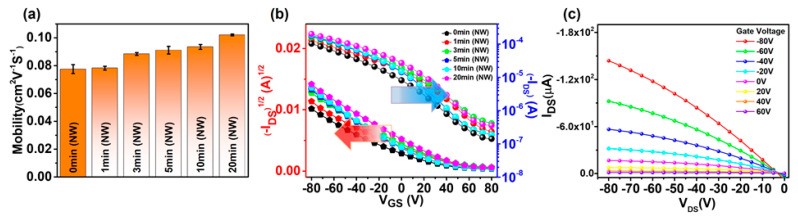
(**a**) Average field-effect mobilities of P3HT-NW film-based organic field-effect transistors (OFETs) as a function of CF vapor annealing time. (**b**) Transfer curves of the corresponding OFETs. (**c**) Output curve of OFETs based on P3HT-NW film annealed with CF vapor for 20 min.

The effects of SVA on the charge transport properties were investigated by measuring the field-effect mobilities of the P3HT-NW films annealed with CF vapor for different times. Figure 7a shows the evolution of mobility over the annealing time. The mobility was gradually increased by 31% (i.e., from 7.8 × 10^−2^ to 10.2 × 10^−2^ cm^2^ V^−1^ s^−1^) as the annealing time was increased from 0 to 20 min. Further increase in the annealing time up to 50 min did not lead to a discernable change in mobility (Figure S7a from the Supplementary Materials). This increase in mobility is attributed to the P3HT nanofibrillar structures formed via strong interchain interactions during CF vapor annealing, which serve as pathways for efficient charge transport within the resulting films. The same trend was observed in mobility variation of pristine P3HT films upon increasing the CF vapor annealing time (Figure S8a from the Supplementary Materials). Transfer and output characteristics of the P3HT-NW-based OFETs exhibit a typical *p*-channel OFET operation in the accumulation mode at negative gate voltage (Figure 7b,c, Figures S7b and S8b).

## 4. Conclusions

In conclusion, we investigated the effects of SVA on the molecular ordering, morphology, and charge transport properties of the P3HT films containing preformed P3HT NWs. SVA using CF vapor greatly facilitated the formation of crystalline P3HT nanofibrillar structures via strong π–π interactions between P3HT chains. In particular, the generation of crystalline P3HT NWs was more prominent in the P3HT films obtained from the solutions exposed to UV irradiation upon CF vapor annealing, probably due to the presence of nucleation sites and small crystallites, compared to pristine P3HT films. It was revealed that the annealing time profoundly influences the molecular ordering, morphology, and charge transport characteristics of P3HT films. As the annealing time was increased up to 20 min, increasingly, the degree of molecular ordering was enhanced and the generation of the P3HT NWs was promoted. As a result, the P3HT-NW-film CF vapor annealed for 20 min exhibited a mobility of ~10.2 × 10^−2^ cm^2^ V^−1^ s^−1^, which is 32% greater than that of the pristine P3HT-NW films (~7.8 × 10^−2^ cm^2^ V^−1^ s^−1^) and 340% greater than that of the pristine P3HT films (~2.3 × 10^−2^ cm^2^ V^−1^ s^−1^). The studies presented here provide new insights into post-deposition processes, such as solvent vapor annealing and thermal annealing, for the fabrication of low-cost flexible high performance organic electronic devices.

## Supplementary Materials

The following are available online at https://www.mdpi.com/2073-4360/12/5/1188/s1: Figure S1, Tapping mode AFM images of spin-coated P3HT-NWs-embedded P3HT thin films annealed by CF vapor for 50 min; Figure S2, Surface roughness of pristine P3HT films annealed with CF vapor different times (0 and 20 min); Figure S3, Normalized UV–vis absorption spectra of spin-coated P3HT-NW and pristine films treatment CF vapor annealed at 1, 3, 5, 10, 20, 30, 40, and 50 min; Figure S4, Normalized UV-vis absorption spectra of spin-coated P3HT-NW films annealed with CF vapor for 30, 40, and 50 min, respectively; Figure S5 Absorption spectra of P3HT-NW films annealed with CF vapor for 0, 1, 3, 5, 10, 20, 30, 40, and 50 min deconvoluted by the Spano analysis; Figure S6, Absorption spectra of pristine P3HT films annealed with CF vapor for 0, 1, 3, 5, 10, 20, 30, 40, and 50 min deconvoluted by the Spano analysis; Figure S7, Average field-effect mobilities and transfer curves of OFETs based on P3HT-NW films annealed with CF for 30, 40, and 50 min; Figure S8, Average field-effect mobilities and transfer curves of OFETs based on pristine P3HT films annealed with CF for 0, 1, 3, 5, 10, 20, 30, 40, and 50 min.
