# Supplementary material for: Effects of Solvent Vapor Annealing on Morphology and Charge Transport of Poly(3-hexylthiophene) (P3HT) Films Incorporated with Preformed P3HT Nanowires
Source: Polymers (Basel). 2020 May 22;12(5):1188. doi: 10.3390/polym12051188 (PMC7284567; doi:10.3390/polym12051188)
Supplement: Supplementary file 1 [file polymers-12-01188-s001.pdf]

Supporting Information

Effects of Solvent Vapor Annealing on Morphology and Charge Transport of Poly(3-hexylthiophene) (P3HT) Films Incorporated with Preformed P3HT Nanowires

Mingu Jang [1], Yang-Il Huh, [1,2,*] Mincheol Chang [1,2,3,*]

[1]Department of Polymer Engineering, Graduate School, Chonnam National University, Gwangju 61186, South Korea

[2]School of Polymer Science and Engineering, Chonnam National University, Gwangju 61186, South Korea

[3]Alan G. MacDiarmid Energy Research Institute, Chonnam National University, Gwangju 61186, South Korea

*Correspondence: yihuh@chonnam.ac.kr (Y.-I.H.); mchang35@chonnam.ac.kr (M.C.); Tel.: +82-62-530-1771 (M.C.)

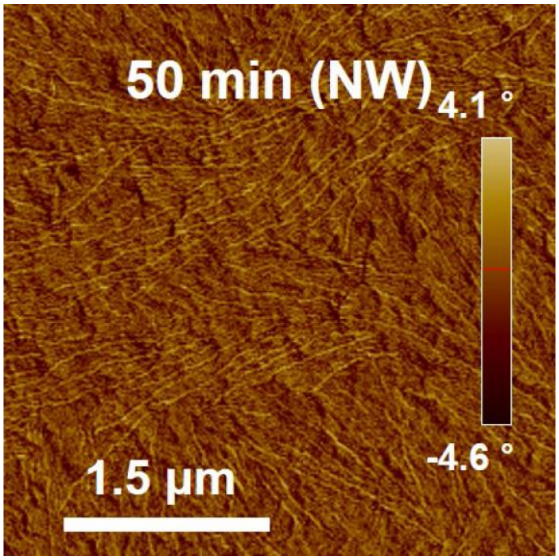

Figure S1. Tapping mode AFM image of a spin-coated P3HT-NW film annealed with CF vapor for 50 min.

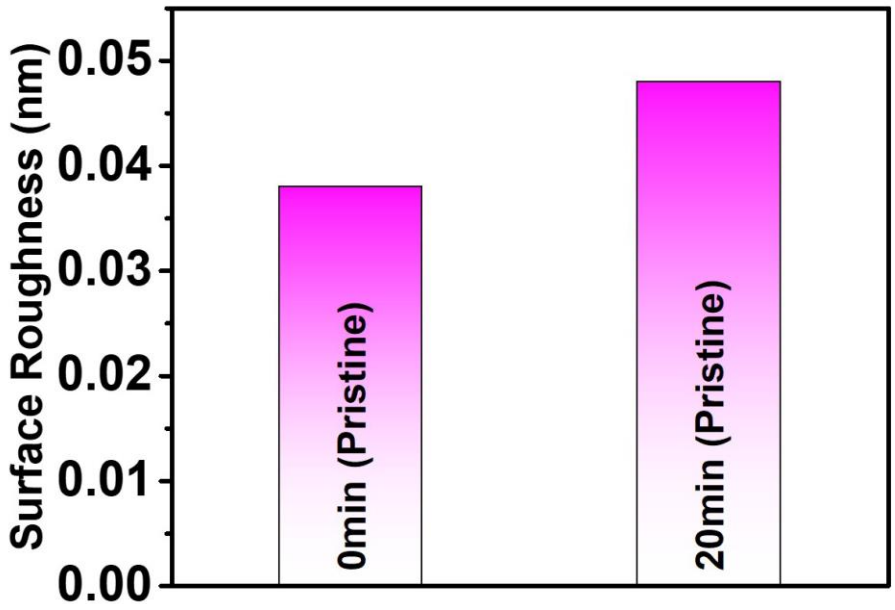

Figure S2. Surface roughness of pristine P3HT films annealed with CF vapor for different times (0 and 20 min).

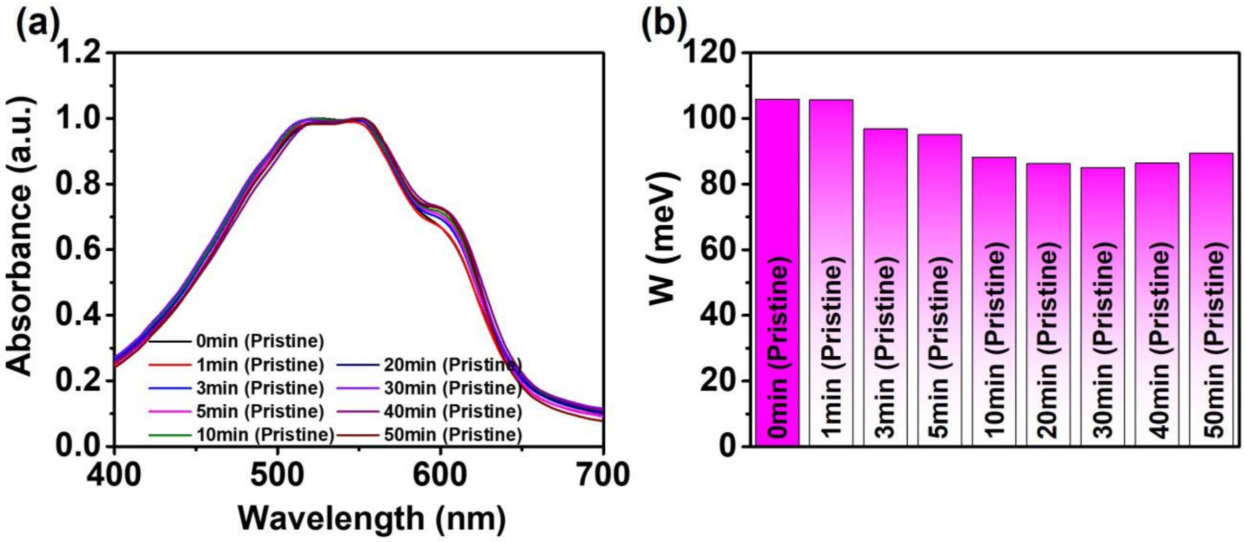

Figure S3. (a) Normalized UV-vis absorption spectra of spin-coated pristine P3HT films annealed with CF vapor for 0, 1, 3, 5, 10, 20, 30, 40, and 50 min. (b) Calculated exciton bandwidth (W) of the corresponding P3HT films.

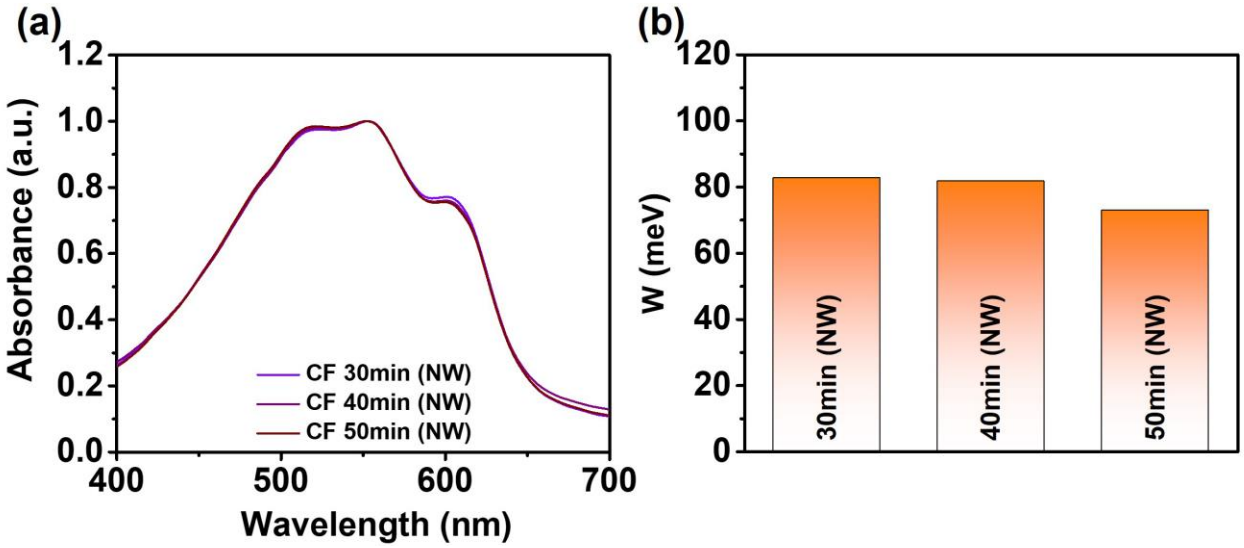

Figure S4. (a) Normalized UV-vis absorption spectra of spin-coated P3HT-NW films annealed with CF vapor for 30, 40, and 50 min, respectively. (b) Calculated exciton bandwidth (W) of the corresponding films.

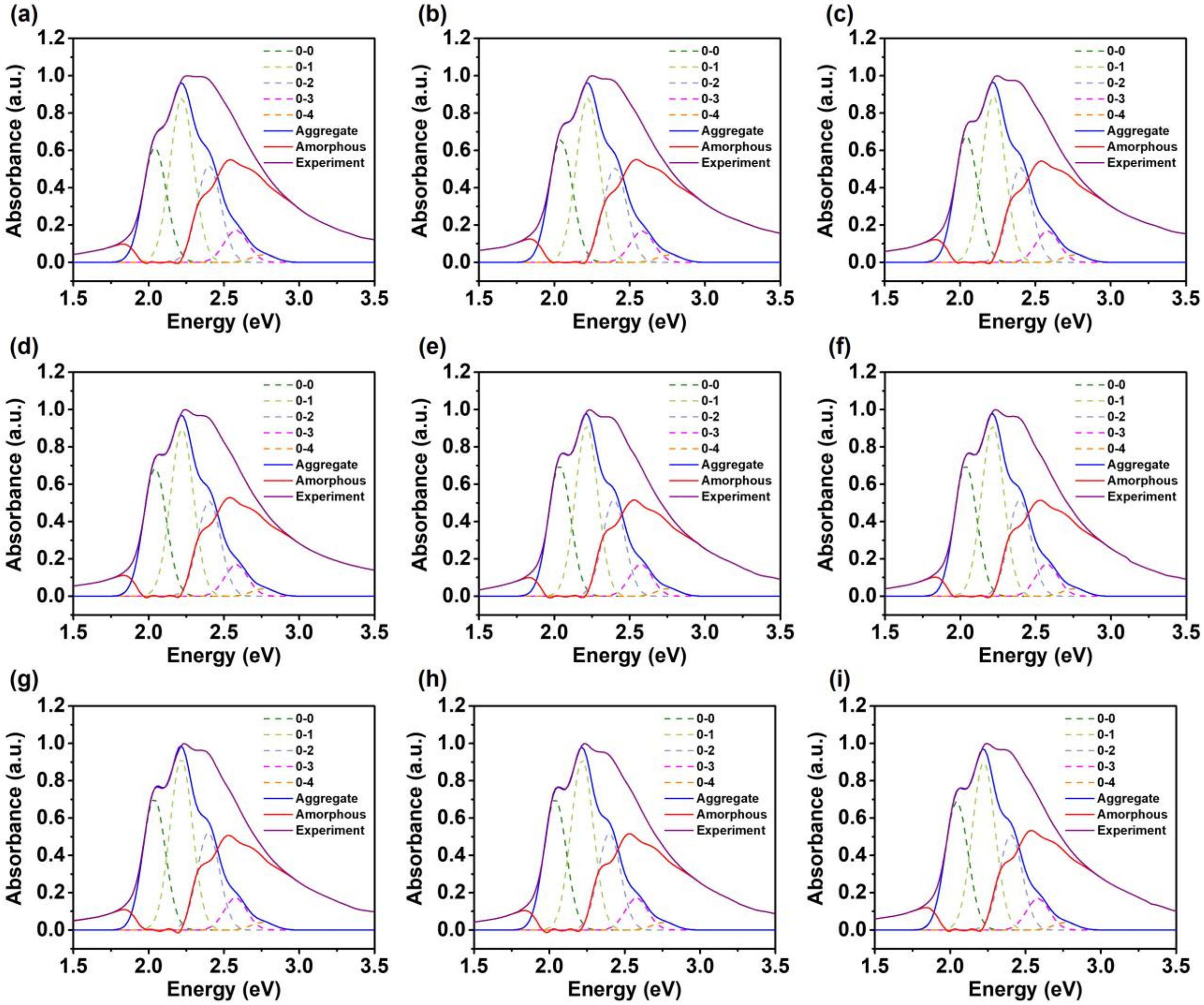

Figure S5. Absorption spectra of P3HT-NW films annealed with CF vapor for (a) 0, (b) 1, (c) 3, (d) 5, (e) 10, (f) 20, (g) 30, (h) 40, and (i) 50 min deconvoluted by the Spano analysis. The red line indicates the spectrum of the amorphous P3HT chains and blue line depicts the spectrum of P3HT aggregates in the films.

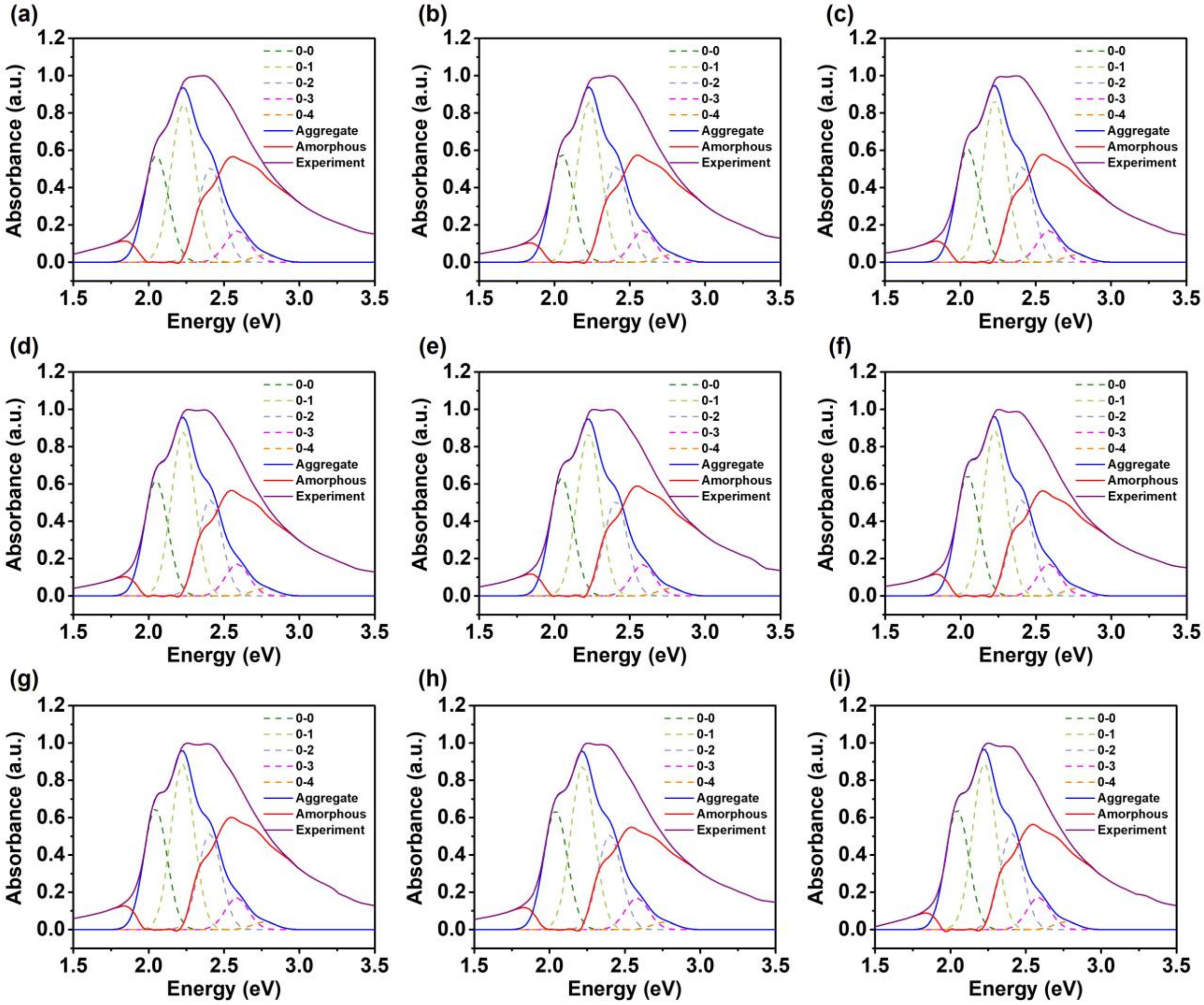

Figure S6. Absorption spectrum of pristine P3HT films annealed with CF vapor for (a) 0, (b) 1, (c) 3, (d) 5, (e) 10, (f) 20, (g) 30, (h) 40, and (i) 50 min deconvoluted by the Spano analysis. The red line indicates the spectrum of the amorphous P3HT chains, and blue line depicts the spectrum of P3HT aggregates in the films.

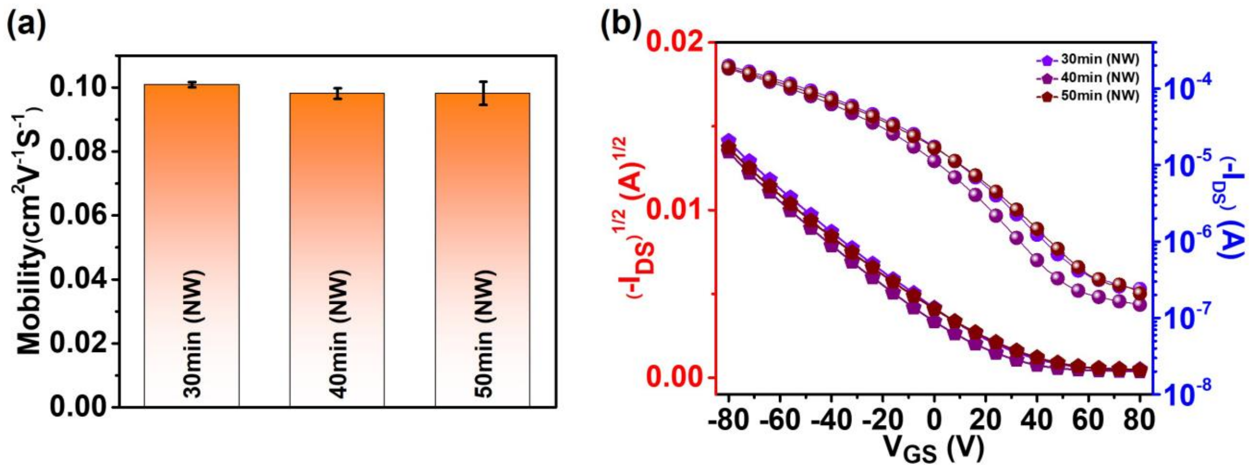

Figure S7. (a) Average field-effect mobilities and (b) transfer curves of OFETs based on P3HT-NW films annealed with CF for 30, 40, and 50 min, respectively.

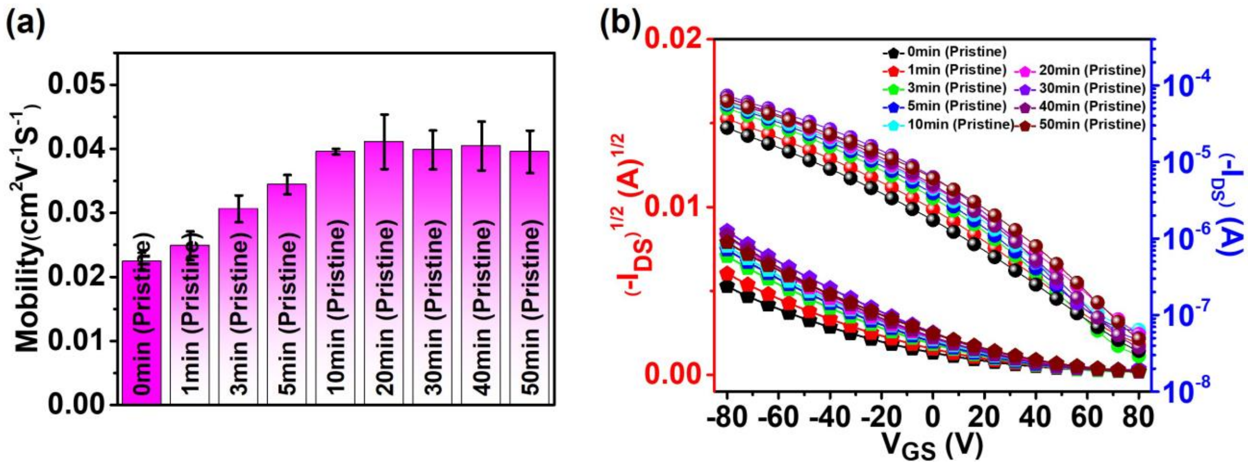

Figure S8. (a) Average field-effect mobilities and (b) transfer curves of OFETs based on pristine P3HT films annealed with CF for 0, 1, 3, 5, 10, 20, 30, 40, and 50 min, respectively.
